# Phenotypic flexibility as key factor in the human nutrition and health relationship

**DOI:** 10.1007/s12263-014-0423-5

**Published:** 2014-08-09

**Authors:** Ben van Ommen, Jan van der Greef, Jose Maria Ordovas, Hannelore Daniel

**Affiliations:** 1 TNO, Zeist, The Netherlands; 2 Leiden University, Leiden, The Netherlands; 3 Sino-Dutch Centre for Preventative and Personalized Medicine, Zeist, The Netherlands; 4 Nutrition and Genomics Laboratory, Tufts University Boston, Boston, MA USA; 5 Technical University Munich, Munich, Germany

**Keywords:** Phenotypic flexibility, Systems biology, Metabolism, Biomarker

## Abstract

Metabolic adaptation to a disturbance of homeostasis is determined by a series of interconnected physiological processes and molecular mechanisms that can be followed in space (i.e., different organs or organelles) and in time. The amplitudes of these responses of this “systems flexibility network” determine to what extent the individual can adequately react to external challenges of varying nature and thus determine the individual’s health status and disease predisposition. Connected pathways and regulatory networks act as “adaptive response systems” with metabolic and inflammatory processes as a core—but embedded into psycho-neuro-endocrine control mechanisms that in their totality define the phenotypic flexibility in an individual. Optimal metabolic health is thus the orchestration of all mechanisms and processes that maintain this flexibility in an organism as a phenotype. Consequently, onset of many chronic metabolic diseases results from impairment or even loss of flexibility in parts of the system. This also means that metabolic diseases need to be diagnosed and treated from a systems perspective referring to a “systems medicine” approach. This requires a far better understanding of the mechanisms involved in maintaining, optimizing and restoring phenotypic flexibility. Although a loss of flexibility in a specific part of the network may promote pathologies, this not necessarily takes place in the same part because the system compensates. Diagnosis at systems level therefore needs the quantification of the response reactions of all relevant parts of the phenotypic flexibility system. This can be achieved by disturbing the homeostatic system by any challenge from extended fasting, to intensive exercise or a caloric overload.

## Introduction

Physiology maintains a well-orchestrated machinery allowing the organism to adapt to the continuously changing environment, of which food takes a major share. We term this organism response capacity “phenotypic flexibility” and this is from our understanding central for overall homeostasis and thus for a healthy life. Processes and mechanisms forming the basis of phenotypic flexibility include substrate fluxes for ATP production and biosynthesis, oxidative stress and inflammatory responses, immune functions as well as DNA repair, apoptosis and others. All of these processes involve multiple molecular mechanisms, interconnected and coordinated by complex regulatory networks which need a real systems approach for the most comprehensive description and in-depth understanding.

Daily meals provide energy pulses which are efficiently absorbed and distributed for immediate needs in ATP production or stored as glycogen or lipids. Possible side effects of these energy pulses are oxidative and inflammatory responses that are ideally quenched by counteracting mechanisms. Stress reactions on the other side require the immediate mobilization of endogenous energy resources with changes in substrate flow for the metabolic needs. Chronic stress conditions may induce adaptation processes that go beyond the limits of normal phenotypic flexibility leading to progressive inflexibility, which in turn contributes to a disease onset. Food components, by excess or by lack in the diet, challenge phenotypic flexibility: Essential nutrients and other food bioactives, when consumed appropriately, play key roles in the mechanisms that maintain phenotypic flexibility, for example as cofactors, while excess of energy, high glucose and fructose intakes or certain trans-fatty acids cause a decline in phenotypic flexibility.

The importance of maintaining flexibility as a key feature to optimal health calls for new research on the relationship between nutrition and health and in particular with the identification of valid “biomarkers of health,” related to the dynamics of regulatory processes. These markers may be found by exploiting the processes and quantify the response reactions to a stress challenge including a “dietary stress” (van Ommen et al. 2008, 2009).

The key example for the loss of phenotypic flexibility is the development of type 2 diabetes with the impaired response to insulin secretion. It usually takes years from an initial impairment of insulin action in peripheral tissues and liver with hyperglycemia, hyperinsulinemia to a progressive loss of β-cell responsiveness and finally complete loss of β-cell mass. Proper diagnosis of phenotypic flexibility in this context can be made by analyzing all relevant changes in accessible compartments (plasma, exhaled air, urine) during an oral glucose tolerance test (OGTT) and define the amplitude of the changes over time in healthy individuals and those with insulin resistance or diabetes (Ho et al. 2013). But this example also illustrates the complex nature of the phenotypic flexibility concept. Hepatic insulin resistance may be caused by fatty liver as a result of adipose tissue insulin resistance with a limited capacity of adipocytes to take up glucose for conversion into fatty acids and triglycerides (Virtue and Vidal-Puig 2010). But hepatic lipid accumulation may also be caused by a reduced biosynthesis of phosphatidylcholine that is needed for export of the lipids via VLDL and/or by an impaired hepatic fatty acid oxidation (Vance 2013). In view of other dietary determinants, a high fructose intake also increases hepatic de novo fatty acid biosynthesis and the development of non-alcoholic fatty liver disease. In contrast to liver, insulin resistance in peripheral tissues is not only caused by lipotoxicity but most likely also involves, in a causative manner, alterations in amino acid homeostasis—mainly of the branched chain amino acids—and overall protein homeostasis.

**Fig. 1:**
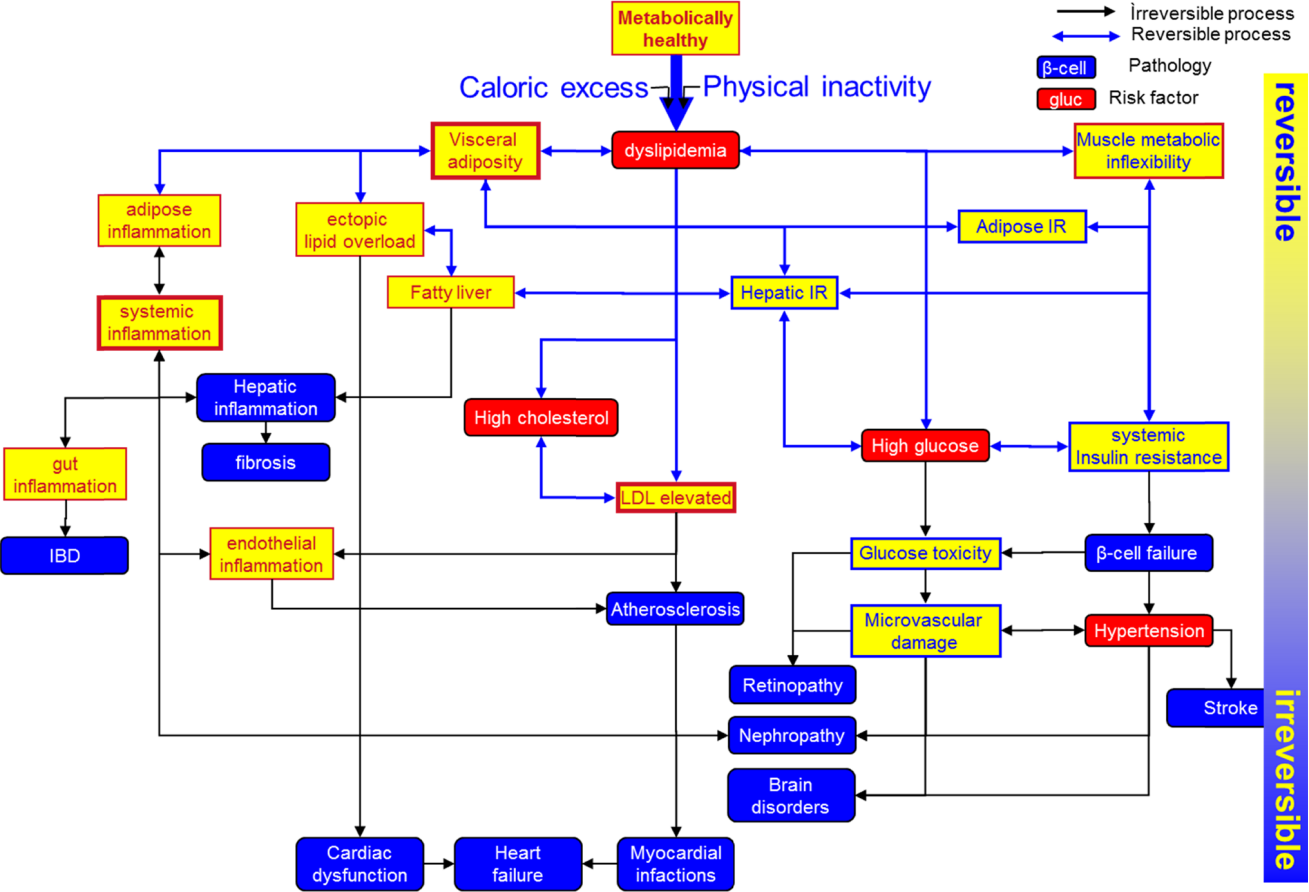
Main processes and events involved in (decrease of) phenotypic flexibility and the resulting (patho-)physiological consequences (adapted from Lusis et al. 2008). Multiple organs and processes are involved in maintaining homeostasis in aspects of metabolism, all of them are interacting with each other and most of them are reversible

Phenotypic flexibility presents a systems view on the complete molecular and physiological “machinery” of stress responses related to metabolic and caloric challenges (Fig. 1). The paragraphs below illustrate the concept of phenotypic flexibility by describing the human phenotypic responses to the three macronutrients glucose, triglycerides and proteins. Together, these examples span from intracellular regulatory mechanisms to whole-body physiology and show the complexity and the need for a systems approach in studying phenotypic flexibility and its application in metabolic health research and systems healthcare.

## Phenotypic flexibility responses to a glucose challenge

### The signaling compensatory response on glucose

Our metabolic system appears not ‘designed’ to deal with large amounts of simple carbohydrate, although dietary intake of large quantities of complex carbohydrates (starch) has been part of human diets throughout recent evolution. Except for some fruits and honey delivering mono and disaccharides in early phases of human evolution, regular consumption of higher amounts of mono- and disaccharides with foods and beverages has become prevalent only in the last few decades. Glucose release from complex carbohydrates such as starch is rate limiting primarily because hydrolysis of glucosidic bonds occurs in the upper small intestine by pancreatic amylase. In contrast, administration of 75 g of d-glucose as during a standard OGTT causes an immediate increase in plasma glucose levels and triggers a large array of compensatory mechanisms. Gastrointestinal hormones such as CCK (Geneontology), GI, GLP1 and PYY increase in response to OGTT (Blaak et al. 2012), which may reflect an “archaic response” of the gastrointestinal tract to a complex meal containing fat, carbohydrates and proteins rather than the artificial condition of an isolated nutrient such as glucose entering the system. Increased plasma glucose, together with the elevated levels of the incretins GIP and GLP1, causes insulin and C-peptide secretion from pancreatic β-cells and a modest inhibition of glucagon secretion from α-cells. Insulin controls glucose disposition in muscle and adipose tissue by recruitment of the glucose transporter GLUT4 into the plasma membrane. The presence of additional GLUT4 allows increased glucose uptake. In the liver, the additional glucose induces glycogen production by changing the activity states of glycogen synthase and phosphorylase downstream of the insulin receptor.

### The metabolic systems response to glucose

Among the most responsive metabolites that increase in plasma after an OGTT are surprisingly primary and secondary bile acids that increase up to 8-fold in concentration compared to fasting (Shaham et al. 2008). Increased bile acid secretion into the gut and rapid absorption into the circulation may be due to the emptying of the gallbladder caused by CCK that is induced by glucose intake. Bile acid stimulation of the TGR5 bile acid receptor and the farnesoid receptor (FXR) in liver may control a variety of metabolic processes that relate to energy expenditure and to plasma triglyceride and cholesterol levels. About 10 different amino acids (branched chain, aromatic and non-proteinogenic amino acids) decline significantly following insulin secretion. They remain below initial levels for more than 2 h although glucose and insulin levels have already returned to baseline. The molecular mechanisms underlying these changes are the insulin-dependent recruitment of amino acid transporters to the plasma membrane (of muscle) allowing increased amino acid delivery to oxidation or to protein synthesis which depends on insulin receptor and mTOR activation. Interestingly, almost all the amino acids that show decreased plasma levels in response to OGTT have been identified in cohort studies as “marker metabolites” of insulin resistance and type 2 diabetes mellitus with increased levels in fasting state (Newgard et al. 2009). Others have associated changes in plasma levels of some of these amino acids as predictive for disease development. This emphasizes the key role of insulin not only in glucose handling but also in amino acid metabolism.

**Fig. 2:**
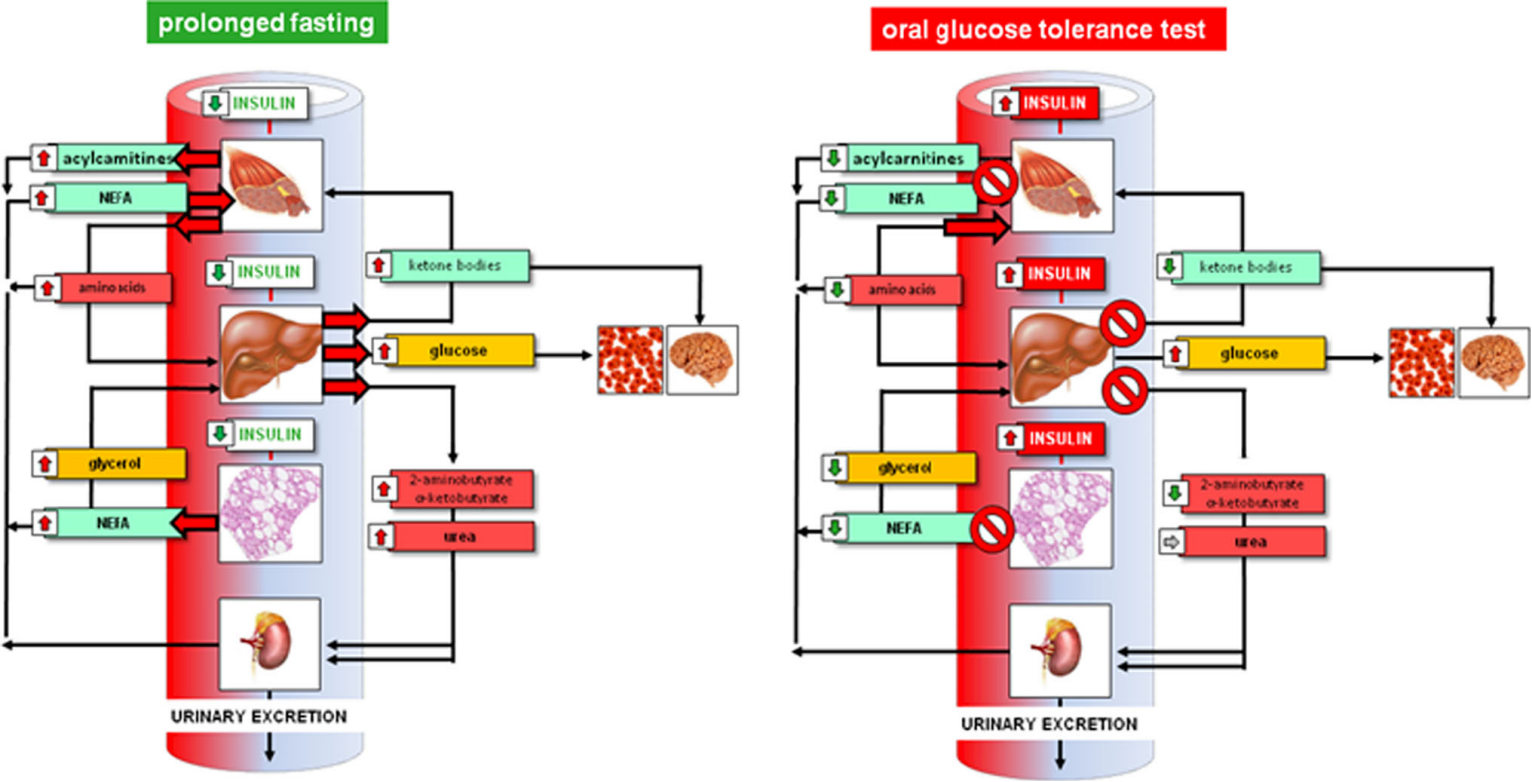
Whole-body metabolic condition and control circuits characterizing a catabolic (fasting) as compared to anabolic/postprandial state with major changes in hormone and metabolite levels which define the etches of a metabolic space and in their time-dependence also serve as a measure of phenotypic flexibility

The OGTT in addition causes a suppression of lipolysis that in turn reduces plasma non-esterified fatty acids (NEFA) and ketone body concentrations. Elevated levels of those metabolites in fasting blood also characterize an insulin-resistant state and type 2 diabetes. Interestingly, there are also significant reductions in plasma acyl-carnitine levels, while free carnitine increases (Krug et al. 2012). Since acyl-carnitines are the products of mitochondrial β-oxidation, these changes in plasma during the OGTT demonstrate the shift from utilization of fatty acids as prime energy substrates during fasting to glucose utilization following the OGTT. After at least 3 h, plasma levels of all measured metabolites have returned to baseline and a steady increase in plasma NEFA, ketone bodies, and amino acid levels occurs again. Those changes, however, are delayed in humans with hyperinsulinemia and insulin resistance indicating a prolonged stay in an anabolic state. In this respect, the delay in clearance of plasma glucose is associated with a delayed return of catabolic marker metabolites to initial values. These changes in plasma metabolite patterns occur in essentially all major metabolic pathways of macronutrient metabolism (glucose, fatty acids, amino acids). Since the release and uptake of these metabolites by organs depend on insulin sensitivity, the OGTT seems particularly suitable to assess phenotypic flexibility of individuals in time-dependence and in organ specificity (Fig. 2).

### Fasting and flexibility

In contrast to the postprandial state, fasting is characterized by a flow of free fatty acids from adipose tissue to muscle and liver, an increase in hepatic ketone body production, and a release of amino acids into circulation mainly for use in hepatic gluconeogenesis. The OGTT, usually performed after an overnight fasting period, reverses these processes within 15–30 min. The time-dependent response and return to homeostasis to an OGTT thus may be taken as a measure of the individual’s flexibility to switch from a catabolic to an anabolic state. Recent studies employing plasma metabolite profiling during the fasting state (Lawton et al. 2008) and after an OGTT in humans (Shaham et al. 2008; Wopereis et al. 2009; Zhao et al. 2009; Spégel et al. 2009; Ho et al. 2013; Krug et al. 2012) have revealed major changes in numerous plasma metabolites that go far beyond the well-known changes in plasma glucose and insulin or NEFA levels.

## Phenotypic flexibility responses to triglyceride challenges

### The liver as gatekeeper in a triglyceride challenge

The hydrolysis of triglycerides in the intestinal lumen and absorption of the constituents is efficient. Lipids are assembled into chylomicrons with the help of apolipoproteins synthesized in epithelial cells and are released into lymph. Because the intestine expresses many enzymes that control mitochondrial, peroxisomal and microsomal oxidation, fatty acids can also be utilized by the enterocyte. Fatty acids play an important role as signaling molecules in the regulation of their own metabolism. The transcription factor PPARα, a member of the superfamily of nuclear receptors, plays an essential role in this regulation. The liver is the master gatekeeper of ingested and de novo synthesized lipids, with far greater capacity than the intestine for storage and maintenance of lipid homeostasis during the transition from fed to fasted states. Both organs, through the assembly and secretion of lipid-containing particles, deliver lipids in the form of lipoproteins to peripheral tissues and cycle between postprandial and postabsorptive states. Oral fat challenges showed dramatic inter-individual differences between the metabolism of triglyceride-rich lipoproteins, attributed to age, sex, genetic factors but also to the characteristic of the fat load or the background diet (Fielding 2011).

### Plasma clearance as flexibility parameter

Importantly, the observed associations between postprandial lipemia-related variables and CVD risk are not primarily due to the levels of chylomicrons released by the gut into the circulation, but rather to the levels of chylomicron remnants remaining in circulation following the fat load and the time required for their total clearance. Apparently, the capacity (flexibility) of the system to return to equilibrium after a metabolic challenge is more relevant to health than the actual deviation from the steady state. Therefore, an impaired catabolism of chylomicron remnants may represent early changes in phenotypic flexibility given their association with cardiovascular disease risk (Chan et al. 2013; Mihas et al. 2011). Besides the measurement of chylomicron remnants or APOB48 (Fujioka and Ishikawa 2009), more recent studies have described the time courses of plasma metabolites and proteins upon different scenarios involving dietary challenges (Beaudoin et al. 2011). However, the relation between traditional and novel biomarkers has not been established.

### Adipose expandability

Following the packaging of dietary fat into chylomicrons and release into lymph, a key tissue for its disposal is adipose tissue and a fundamental element in phenotypic flexibility of the adipose tissue is its expandability. The failure in proper adipose tissue expansion rather than obesity per se is a key factor by which a positive energy balance is linked to type 2 diabetes as well as other metabolic diseases (Virtue and Vidal-Puig 2010; Medina-Gomez et al. 2007). Once the individual’s capacity to store additional fat in the adipocyte is reached, lipids begin to accumulate in other tissues such as liver and muscle leading to harmful consequences (Virtue and Vidal-Puig 2010).

### Circadian rythm

The circadian rhythm is one of the best established mechanisms of phenotypic flexibility that has been ingrained in all living things in order to maintain synchronicity with the pace defined by the rotation of the Earth around its axis. Based on data from studies determining the circadian metabolomic changes, it has become evident that the lipid metabolome is among the most modulated pathways, underscoring the importance of lipid metabolism, including dietary fat and relevance of maintaining this pathway attuned with the environmental changes driven by the daily rhythms of nature. (Eckel-Mahan et al. 2012; Dallmann et al. 2012).

### The inflammatory response

A postprandial inflammatory response to triglicerides (Giuseppe Derosa et al. 2009) but also to glucose (Derosa et al. 2010) or a mixed meal challenge has been described. Yet, a thorough nutrigenomic examination of the inflammatory response resulted in only subtle and minor effects (Pellis et al. 2011; Wopereis et al. 2013). Apparently, in healthy subjects, phenotypic flexibility mechanisms prevent the onset of a strong inflammatory reaction. Interestingly, many studies reporting on a postprandial inflammatory response had no proper control group, confounding the conclusions (for a discussion see (Wopereis et al. 2013). Yet, this area needs further attention, as many relevant but controversial mechanisms have been identified in animal models but still need to be validated for human translation, such as lipid-based leaky gut systemic LPS inflammation (Liang et al. 2014), differences between saturated and unsaturated hepatic inflammation triggers (Lichtenstein et al. 2010). Obviously, chronic caloric overload produces a metabolic-inflammatory response of which the mechanisms are being unraveled (Osborn and Olefsky 2012), but the acute inflammatory response needs further attention as the molecular tools to study this become available.

## Phenotypic flexibility responses to protein challenges

### The importance of amino acid homeostatic control

Dietary protein intake has been fundamentally important for the development of the human species because the amino acid substrates for protein synthesis do not have to be synthesized de novo. Increased protein intake not only allowed the body length to increase in human evolution but was also a key determinant in brain development (Laus et al. 2011). Yet, as protein synthesis cannot be increased infinitely, the current luxury supply of dietary protein requires that substantial quantities of amino acids are oxidized and used as energy substrates. Amino acids contribute on average with 15 to 20 % to fuel oxidation. However, a consequence of amino acid oxidation is the increased release of ammonia which needs to be detoxified by the hepatic urea cycle followed by elimination through the kidneys. In addition, the surplus of methionine and cysteine in the diet provided in larger quantities with animal protein sources increases of sulfate excretion via kidney with net effects on extracellular acidification and increased renal acid elimination. The daily turnover of amino acids exceeds that of lipids by around 30 % and is almost two-fold higher than that of glucose.

Plasma homeostasis of amino acids is most important because numerous individual amino acids have distinct metabolic roles such as neurotransmitters (i.e., glutamate, glycine) or precursors thereof (GABA from glutamate, or catecholamines from tyrosine) or contribute to a variety of synthetic pathways or other regulatory processes. A prime example is nitric oxide (NO) which is derived from arginine and acts as a signaling molecule in numerous processes (Hoang et al. 2013). It has been proposed that dietary arginine is converted into citrulline in the intestine which prevents arginine plasma levels to overshoot in the absorptive phase which would lead to an increased NO production with numerous downstream effects for example on the vasculature (Kaore et al. 2013). Only small quantities of portal blood citrulline are taken up during liver passage, but the kidney extracts most citrulline and converts it back to arginine. Liver extraction of other amino acids delivered through portal blood is substantial with the exception of the branched chain and aromatic amino acids that consequently show the most impressive increases in peripheral blood after protein intake.

### Signaling control of amino acid homeostasis

Amino acid homeostasis is thus tightly regulated at all levels (absorptive, anabolic, catabolic, protein synthesis or degradation). Protein turnover as the sum of degradation and synthesis is subject to regulation by numerous hormones. Insulin reduces protein breakdown and promotes protein synthesis when plasma insulin levels rise and amino acid levels also increase following protein intake. Under these conditions, insulin has the strongest effects on protein balance compared to all other hormones including growth hormone/IGF, epinephrine, testosterone or thyroid hormones. In contrast to insulin, stress hormones such as glucagon, glucocorticoids and catecholamines strongly increase protein breakdown but seem also to be able to modestly increase protein synthesis rate.

In contrast to carbohydrate-rich meals, protein-rich meals cause glucagon secretion, which also occurs in the presence of dietary carbohydrates with a simultaneous increase in plasma insulin. Although some amino acids can effectively elicit insulin secretion (for example arginine), the totality of circulating amino acids in the absorptive phase promotes a substantial glucagon release. This counteracting effect of amino acid on insulin secretion prevents a hypoglycemia during high protein, but low carbohydrate intake. Glucagon increases hepatic gluconeogenesis from amino acids allowing under high protein intake the maintenance of plasma glucose levels and reserves of glycogen. Yet, the clearance of the amino acids from plasma in the postprandial state is increased in the presence of insulin. In diabetes, removal of amino acids from plasma seems delayed and gluconeogenesis from amino acids in liver seems enhanced by the lack of proper suppression by insulin, thus contributing to hyperglycemia. In view of the effects of insulin on protein synthesis in the presence of elevated plasma amino acids during high protein intake, the concerted action of the insulin and mTOR signaling comes into play. mTOR comprises two protein complexes TORC1 and TORC2. mTORC1 (mammalian TORC1) functions as an environmental sensor to promote anabolic and inhibit catabolic cellular functions. It phosphorylates and activates protein kinases S6K1 and S6K2 with the substrate ribosomal protein S6, a component of the 40S ribosome and of 4E-BP1 promoting ribosome genesis and protein synthesis. Intracellular amino acids and among them leucine are sensed by GTPases and activate mTORC1. The downstream target of the insulin receptor signaling pathway Akt inhibits the TSC2 protein and allows Rheb-GTP to accumulate and to activate mTORC1 and thus amplifies the amino acid dependent activation. On the other hand, AMP-kinase is a sensor of the cellular energy status and exerts inhibitory effects on the mTOR system. Interestingly, exercise also enhances mTOR activity and thus promotes metabolic adaptation to nutrients and exercise in skeletal muscle. However, mTORC1 signaling can attenuate Akt activation through a negative feedback mechanism. Inhibitory phosphorylation of the insulin receptor substrate 1 and the mTORC2 component Rictor by mTORC1 and S6-kinase suppresses the ability of insulin to stimulate Akt as a key effector of insulin signaling. Amino acid induced mTORC1 signaling can decrease insulin sensitivity, and this can be observed in all metabolically important tissues examined. In this respect, it is not surprising that amino acid homeostasis is impaired in humans with the metabolic syndrome. That obesity affects amino acid metabolism is known since 1969 when it was first described that plasma levels of branched chain amino acids (BCAA) are elevated in obesity (Felig et al. 1969). It was recently shown that these changes in plasma BCAA are also found in obese children and that they predict future development of insulin resistance (McCormack et al. 2013). Numerous studies employing metabolite profiling in insulin-resistant humans or patients with type 2 diabetes (fasting plasma) have identified predominantly amino acids such as the BCAA and their degradation products (Newgard et al. 2009), glycine, citrulline and proline but also aminoadipic acid from lysine degradation as marker metabolites (Menni et al. 2013). However, there are currently no studies that demonstrate impairments in phenotypic flexibility related to amino acid or nitrogen handling in insulin resistance or diabetes when the metabolic system is challenged with larger quantities of dietary protein.

Taken together, a high protein intake—whether acute or chronic—has complex effects on the metabolic system. It reduces the metabolic adaptation to insulin action, and forces amino acids to be used as energy substrates thus reducing the need to burn lipids and carbohydrates which may promote insulin resistance and diabetes by their glucotoxicity and lipotoxicity. In addition, increased gluconeogenesis in insulin-resistant state further enhances hyperglycemia. The need for elimination of the nitrogen surplus challenges hepatic urea production and the renal elimination. In addition, sulfur derived from methionine and cysteine intake challenges the acid–base balance.

## Conclusion and perspective

The above examples of carbohydrate (glucose), lipid (triglycerides and fatty acids) and protein related phenotypic flexibility all trigger acute and complex stress response mechanisms of which optimal functioning is essential for maintenance of metabolic health. Quantification of aspects of these stress response reactions is informative on health status and can be exploited as “biomarker of health.” In this sense, the OGTT is well established and discussed above, but research on triglycerides (Blaak et al. 2006; Krug et al. 2012; Musso et al. 2009), protein (Fernandes and van de Kamer 1968; Bosch et al. 1984) and various combinations of glucose, triglycerides and proteins, either as mixture of the pure components (Pellis et al. 2011; Wopereis et al. 2013) or as mixed meal challenges (Phillips et al. 2010; van Dijk et al. 2012) has been published. Also, mathematical methods to analyze the stress response curves are being developed (Vis et al. 2014). Each of these “stress tests” has pro’s and con’s in terms of practical use vs value for mechanistic interpretation. Apart from the OGTT, the “standardized phenotypic flexibility assay” now emerges as 75 g of glucose, 60 g of Palm oil and 30 g of protein.

All of these acute response reactions illustrate a number of characteristics of phenotypic flexibility:

1. All processes result from an acute perturbation and show a time course of stress response in the hour scale, at first glance simple (“glucose curve”) but intrinsically complicated, spanning major parts of metabolic-inflammatory control;
2. All processes are interdependent and can act via multiple connections and feedback mechanisms;
3. Failure of a specific process in this phenotypic flexibility network may be compensated by other processes, but eventually lead to harmful adaptive processes;
4. Interestingly, the “site of failure” does not need to be the site of emerging pathology. Impaired adipose expandability will result in ectopic lipid, inflammation and hepatic/vascular disease.
5. Quantification of the molecular stress response reactions after a standardized metabolic/caloric perturbation such as the OGTT (Krug et al. 2012), standardized mixed meal challenges (Pellis et al. 2011) or even Big Macs (Bondia-Pons et al. 2014) (e.g., by plasma metabolomics) reveals a detailed picture of the events and can be used as biomarker and diagnostics of phenotypic flexibility.

In contemplating health from a phenotypic flexibility systems perspective, prevention of disease onset and the role of diet in maintaining health become tangible and concrete as many nutrients play a crucial role in the mechanisms underlying the response pathways. For example, dietary factors such as fructose but also betaine, choline, carnitine and others play an important role both in onset and prevention of fatty liver (de Wit et al. 2012; Malaguarnera et al. 2010; Noland et al. 2009; Rutlegde and Adeli 2007; Fischer et al. 2010). Oxidative stress and inflammatory stress responses also rely on diet-derived factors such as the eicosanoids as products of essential fatty acids (Wopereis et al. 2013). We now realize that, in general, dietary factors have roles that are mirror images of those provided by drugs: The first optimize and facilitate processes (salutogenesis, health promotion), while the latter mainly inhibit processes (disease management), which by definition does not match with the concept of strengthening or improving phenotypic flexibility.

Finally, we would like to emphasize that phenotypic flexibility as defined above is embedded in a larger system. It is part of a much wider flexibility system determining and maintaining optimal health, which can only be understood by integrating physiology and psychology, and also taking into account the social environment. This bio-psychological domain focuses on psycho-neuro-endocrinology instead of neuro-endocrinology alone. Visceral adiposity is not only associated with a blunted metabolic flexibility, but also with reduced flexibility in neuroendocrine and cognitive responses (Jones et al. 2012). From the psychological perspective, the relationship with metabolic/inflammatory health is becoming apparent. For example, social support is related to changes in cardiovascular, neuroendocrine and immune function, optimism has been associated with immune up-regulation, and adaptive coping is linked with reduced cortisol output (see (Steptoe et al. 2009) and references). Here, the well-known concept in psychology of resilience nicely aligns with phenotypic flexibility.

Secondly, flexibility has different time dimensions. As described above, phenotypic flexibility responses to acute challenges occur with timescales of minutes to hours while chronic adaptation processes and allostatic processes, as the consequence of all efforts to maintain homeostasis and flexibility that result in pathologies, usually span months to decades. Some of these adaptive processes and their health consequences have already been mentioned above.

The emphasis for life science research has to be aligned with the improved insights related to the new concepts and definitions of health. Studies need to focus more on system response monitoring, measuring the dynamics of regulatory processes in a much broader context.
